# Metabolic strategies in hypoxic plants

**DOI:** 10.1093/plphys/kiae564

**Published:** 2024-10-24

**Authors:** Hans van Veen, Paolo Maria Triozzi, Elena Loreti

**Affiliations:** Groningen Institute for Evolutionary Life Sciences, University of Groningen, 9747AG Groningen, The Netherlands; PlantLab, Institute of Plant Sciences, Sant’Anna School of Advanced Studies, 56010 Pisa, Italy; Institute of Agricultural Biology and Biotechnology, CNR, National Research Council, 56124 Pisa, Italy

## Abstract

Complex multicellular organisms have evolved in an oxygen-enriched atmosphere. Oxygen is therefore essential for all aerobic organisms, including plants, for energy production through cellular respiration. However, plants can experience hypoxia following extreme flooding events and also under aerated conditions in proliferative organs or tissues characterized by high oxygen consumption. When oxygen availability is compromised, plants adopt different strategies to cope with hypoxia and limited aeration. A common feature among different plant species is the activation of an anaerobic fermentative metabolism to provide ATP to maintain cellular homeostasis under hypoxia. Fermentation also requires many sugar substrates, which is not always feasible, and alternative metabolic strategies are thus needed. Recent findings have also shown that the hypoxic metabolism is also active in specific organs or tissues of the plant under aerated conditions. Here, we describe the regulatory mechanisms that control the metabolic strategies of plants and how they enable them to thrive despite challenging conditions. A comprehensive mechanistic understanding of the genetic and physiological components underlying hypoxic metabolism should help to provide opportunities to improve plant resilience under the current climate change scenario.

## Introduction

Plants require oxygen, primarily to support their energetic demands. Although plants frequently experience hypoxia, it poses serious challenges to how they function. Plants experience hypoxia due to limited aeration in compact tissues or reduced gas diffusion from the environment caused by a flooding event. Limited aeration caused by flooding is also associated with poor light conditions and low photosynthesis, which strongly reduces the availability of fixed carbon. Under poorly aerated conditions, plants exploit several metabolic strategies to cope with hypoxia and limited carbon and to optimize growth (Bailey-Serres and Voesenek 2008; Loreti et al. 2016; Loreti and Perata 2020; Dalle Carbonare et al. 2023).

**Figure 1.**
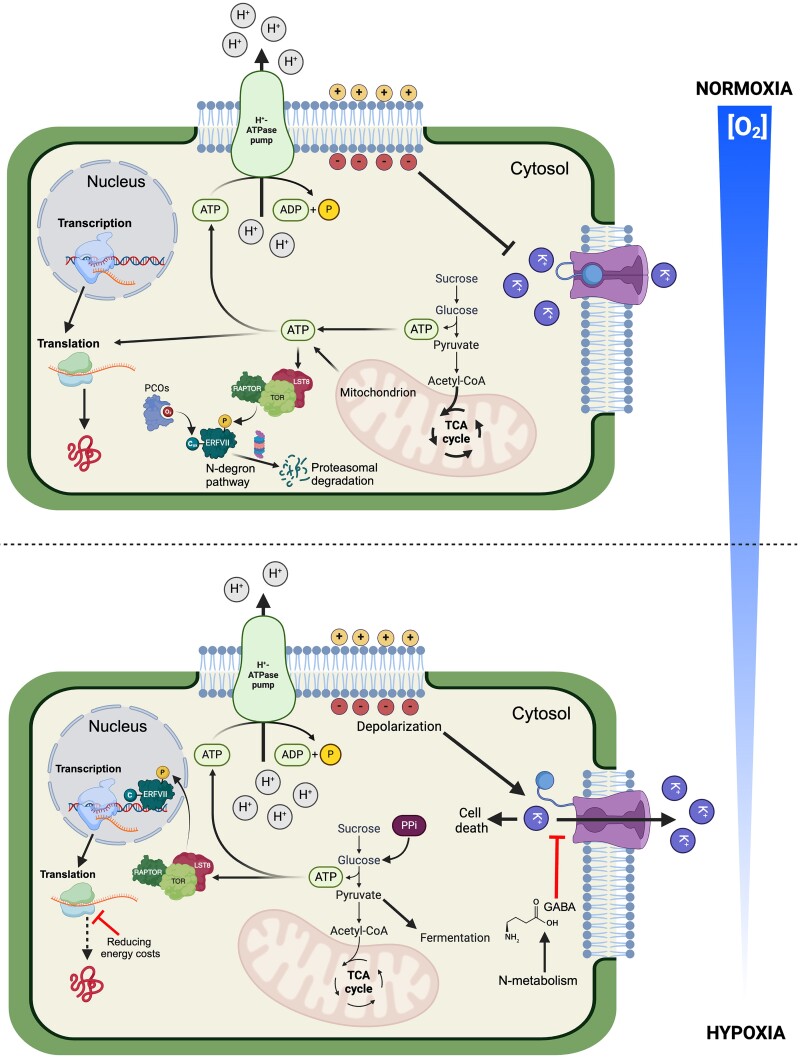
Toward an energy-efficient plant cell during hypoxia. Energy costs are strongly decreased by the inhibition of translational and a drop in H^+^-ATPase activity. Low H^+^-ATPase results in a reduction in the membrane potential which normally leads to potassium losses out of the cell through outward rectifying channels. The resulting loss of K^+^ triggers programmed cell death. Therefore, for H^+^-ATPase to operate within minimal ATP consumption, the increased GABA levels under hypoxia de-sensitize K^+^ efflux upon a drop of inner membrane potential. PPi from residual biosynthetic activity can also be leveraged in sucrose breakdown and glycolysis to maximize ATP output. Aerobic respiration in the mitochondria generates most of the energy (ATP) needed for cellular metabolism. ERFVII transcription factor genes are continuously expressed; however, their stability is undermined by the activity of PCOs. These enzymes, in an oxygen-dependent process, oxidize the N-terminal cysteine residue, directing the ERFVII proteins to the proteasome. Under normoxic conditions, oxygen activates PCOs, which are enzymes that oxidize the N-terminal cysteine residue in proteins that have a Cys residue following the removal of the N-terminal methionine. When hypoxia causes inefficient carbohydrate metabolism and reduces ATP production, the lower ATP content reduces TOR activity. This decrease in TOR activity weakens the induction efficiency of HRGs by ERF-VIIs. Created in BioRender. Triozzi et al. (2024)BioRender.com/w48h170.

When oxygen availability is limited, mitochondrial respiration is impaired, since NADH can no longer be oxidized through the mETC. The inability to regenerate NAD^+^ prevents biochemical reactions in glycolysis and the tricarboxylic acid (TCA) cycle, where it is required as a cofactor. The proton motive force driving ATP synthesis cannot be maintained, leading to a rapid decrease in ATP levels. Therefore, during hypoxia an alternative for the regeneration of NAD^+^ is needed. A shift toward fermentation, through the production of ethanol or lactic acid from pyruvate, enables this reoxidation of the NADH to NAD^+^. Fermentation provides sufficient NAD^+^ to maintain glycolysis with a corresponding low ATP yield. Without mitochondrial respiration, a large portion of the ATP potential of sugars remains untapped, resulting in a severe energy limitation (Fig. 1; Perata and Alpi 1993; Loreti et al. 2016).

Despite its dramatic impact on plant physiology and survival, hypoxia does not always lead to a strong ATP and redox crash. For example, upon anoxia treatment, the shoots of rice coleoptiles *Potamogeton distinctus* and *Sagittaria pygmaea* maintain adenylate levels and energy charge for days (Ishizawa et al. 1999). Similarly, in isolated Arabidopsis leaf sections, the NAD^+^/NADH ratio returned to control levels within several hours of hypoxia treatment (Wagner et al. 2019).

Hypoxia is also experienced by plants at different stages of development and in specific tissues under optimal growing conditions. Indeed, hypoxic niches, found in the shoot apex and lateral root primordia, are essential for the timely and active development of leaves and lateral roots (Shukla et al. 2019; Weits et al. 2019). Oxygen levels also fluctuate over the day–night cycle inside young developing leaves and are integrated with metabolism and growth regulation (Triozzi et al. 2024). Thus, hypoxia can be perfectly integrated with plant growth and developmental processes, resulting in an essential cue for plant functioning (Weits et al. 2021).

Successful acclimation to flooding often requires the development of aerenchyma, adventitious roots, radial oxygen loss barriers, or shoot elongation to improve oxygen movement to submerged parts of the plant and prevent hypoxia (Dalle Carbonare et al. 2023). Some species employ an alternative strategy by stopping growth and development in an attempt to maintain resources to regrow once the flood water recedes (Hattori et al. 2011). Additionally, plants have a sophisticated system of sensing oxygen, thus triggering a corresponding transcriptional and developmental response, which for the transcriptional activation of fermentation enzymes, as well for many other hypoxia-regulated genes, requires the stabilization of ethylene response factors belonging to group VII (ERFVIIs) (see Box 1; Gibbs et al. 2011, 2014; Licausi et al. 2011; Weits et al. 2014; White et al. 2017; Hartman et al. 2019; Loreti and Perata 2023; Zubrycka et al. 2023).

Box 1. **Oxygen sensing in plants.**

In plants, transcription factors belonging to group VII of the Ethylene Response Factors (ERFVIIs) are required for transcriptional activation of HRGs, which include genes encoding enzymes required for the fermentation machinery (Loreti and Perata 2023). ERF-VIIs feature a conserved N-terminal motif that allows destabilization in the presence of oxygen, and they are stable only under low oxygen conditions via the N-degron pathway (Gibbs et al. 2011; Licausi et al. 2011). In the N-degron pathway, enzymatic cleavage of the N-terminal methionine results in an exposed cysteine, which undergoes oxidation by the action of PLANT CYSTEINE OXIDASES (PCOs) under normoxia (Weits et al. 2014). An arginine residue is then added to the oxidized Cys residue by an ARGINYL tRNA TRANSFERASE (ATE), facilitating proteasomal degradation via the N-recognin E3 ligase PROTEOLYSIS (PRT6) (Weits et al. 2014; White et al. 2017). Under hypoxia, PCO enzymes fail to oxidize the Cys at the N terminus, leading to the stabilization of ERFVIIs, which in turn triggers the transcription of HRGs and results in a transcriptional output dependent on the oxygen availability (Zubrycka et al. 2023).

Proteasomal degradation of ERFVIIs also requires nitric oxide (NO), although the exact mechanism remains unclear (Gibbs et al. 2014). NO dependence of ERF-VIIs allows for HRG regulation, also irrespectively of the presence of oxygen (Gibbs et al. 2014).

Under poor aeration, the gaseous hormone ethylene tends to accumulate in the plant (Sasidharan et al. 2018). In Arabidopsis, ethylene promotes PHYTOGLOBIN 1 (PGB1), which scavenges NO and helps to stabilize ERFVIIs (Hartman et al. 2019). ERFVII activity as transcription factors is modulated by calcium, energy, and retrograde signaling, indicating the tight regulation of HRGs and integration with many pathways and processes (Giuntoli and Perata 2018; Cho et al. 2021; Barreto et al. 2022; Dalle Carbonare et al. 2023; Fan et al. 2023; Kunkowska et al. 2023).

In this review, we focus on the metabolic dilemmas encountered by plants to deal with flooding and reduced oxygen levels. These include the challenges of maintaining energy homeostasis and a high sugar supply, strategies when there are limited sugars for fermentation, and the regulation of fluxes through glycolysis and fermentation to regulate plant growth.

## Hypoxic metabolism to maintain cellular homeostasis

The reduced energy efficiency of sugar metabolism without oxygen makes energetic homeostasis very challenging (Fukao and Bailey-Serres 2004). A key to achieving a new energy balance is to reduce protein synthesis, which is the highest cost of cellular ATP (Edwards et al. 2012). However, there is an increase in de novo synthesis of enzymes involved in hypoxic metabolism, including alcohol dehydrogenase (ADH) and pyruvate decarboxylase (PDC) (Chang et al. 2000; Juntawong et al. 2014). When the capacity of plants to produce these enzymes is prevented either through an anoxic shock or protein synthesis inhibitors, it compromises their survival without oxygen, further highlighting the importance of acclimatizing the metabolism (Chang et al. 2000).

Leveraging pyrophosphate (PPi) as an alternative to ATP to increase ATP production is another way of establishing homeostasis (Mustroph et al. 2014b; Atwell et al. 2015). This involves 4 enzymes that are invariably induced upon flooding or hypoxia. In short, sucrose breakdown via sucrose synthase instead of invertase has a net savings of 1 mole of ATP at the cost of PPi, which improves performance during flooding and hypoxia (Bieniawska et al. 2007; Santaniello et al. 2014). Additionally, fructose-6-phosphate 1-phosphotransferase (PFP) and plastidic pyruvate, orthophosphate dikinase catalyze reversible reactions in glycolysis and gluconeogenesis, which also use PPi instead of ATP as the energy source (Gutiérrez-Luna et al. 2018). However, the direction of the reactions under limited aeration is still unclear, and the role of tolerance has not been clearly established (Mustroph et al. 2014b). Lastly, the energy from PPi hydrolysis can be used to pump protons into the vacuole (Ferjani et al. 2012). By making use of the PPi-dependent pathways, the stoichiometry of sucrose breakdown leads to a doubling of the ATP yield of glycolysis (Huang et al. 2008). PPi in the cell is formed during polymer synthesis, and sustained use of PPi-mediated glycolysis needs to be coupled to growth, which might limit its role in quiescent and stressed cells (Atwell et al. 2015). The bidirectional nature of fructose-6-phosphate 1-phosphotransferase and plastidic pyruvate, orthophosphate dikinase combined with an array of physiological contexts enables many variations in flux through these pathways, which clouds the interpretation of their exact role.

Where enhanced glycolytic efficiency and lower protein synthesis costs directly relate to energy homeostasis, metabolic and transcriptomic profiling of plants exposed to hypoxia also typically show an increase in alanine, GABA, and succinate abundance (Narsai et al. 2011; Mustroph et al. 2014a; Coutinho et al. 2018). Although the functional significance and mechanisms behind their accumulation is not fully understood, a range of explanations has been given that each would contribute to energy homeostasis.

Alanine can be formed from pyruvate by the transfer of the amine group from glutamate, which then becomes an oxoglutarate. This is a bidirectional reaction catalyzed by hypoxia-induced alanine aminotransferase. Unlike the conversion of pyruvate into either ethanol or lactate, alanine production from pyruvate does not oxidize NADH and therefore does not contribute to a redox balance. On its own, the functional significance is not readily apparent, nor is the direction of the reaction, since alanine can also be formed from GABA. GABA is a nonproteogenic amino acid that is produced through the decarboxylation of glutamate. The enzyme GABA-T transfers the amine group from GABA to oxoglutarate, pyruvate, or glyoxylate to yield glutamate, glycine, or alanine, respectively. The resulting succinic semi-aldehyde is further converted to succinate. Succinate tends to accumulate under hypoxia because the reaction to fumarate by succinate dehydrogenase, which is bound to cytochrome complex II, does not occur under hypoxia (Rocha et al. 2010; António et al. 2016).

The transferase activity of alanine aminotransferase and GABA-T, combined with the action of GABA decarboxylase, allows carbon backbones to move back and forth between the organic acid and the amino acid pools, resulting in a flexible exchange of metabolites between C and N metabolism. Additionally, it provides an opportunity for oxoglutarate to bypass 2 TCA cycle steps. This is in line with the observations that through the TCA cycle, the fluxes rarely operate in a circular fashion but depend on the demands and conditions of the cell (Sweetlove et al. 2010). Isotope labeling has demonstrated significant movement through the pathway from glutamate to GABA and then to alanine and succinate (António et al. 2016). Stoichiometrically, this bypass has no clear benefits in terms of fixed carbon maintenance, NAD^+^/NADH, or ATP generation. However, glutamate could also be sourced from the existing amino acid pool instead of from oxoglutarate via the GOGAT pathway. This would allow the transfer of compounds from N metabolism to C metabolism, through which a rich pool of amino acids could act as a buffer to absorb hypoxic shocks to metabolism.

The functional implications of Ala and GABA metabolism during hypoxia are still unclear; however, GABA also acts as a signaling molecule for potassium homeostasis and the corresponding electrophysiological balance. Plant cells maintain a negative membrane potential, driven by the plasma membrane H^+^-ATPase. However, the limited energy availability prevents H^+^ export, leading to membrane depolarization. To maintain a negative cellular charge, outward rectifying K^+^ channels (GORKs) open in an attempt to reinstate the membrane potential (Wang et al. 2016). With limited H^+^-ATPase activity, this leads to dramatic loss of potassium and a drop in cytosolic pH (Felle 2005; Mugnai et al. 2011; Wang et al. 2016). Low cytoplasmic potassium levels lead to the inhibition of caspase-like proteases and endonucleases that trigger cell death (Demidchik et al. 2010). However, high GABA levels appear to limit GORK opening upon depolarization. This enables the cell to maintain relatively high K^+^ levels and the associated viability but at a lower plasma membrane potential (Wu et al. 2021). This new steady state (Fig. 1) lowers the costs of maintaining the membrane potential at the cost of less opportunity for transport and growth.

Ultimately, glycolysis dependent on ATP generation, leveraging the PPi pool, limited protein synthesis, and altered amino acid metabolism to regulate pH and stabilize the electrophysiology do not provide a long-term solution. With a finite sugar supply and finite PPi sources, growth is impossible. The abovementioned acclimation (Fig. 1) is thus best suited to a quiescence strategy to withstand short but harsh flood events.

## Providing sugars to fuel fermentation

A lack of oxygen is a serious challenge to maintaining homeostasis and may compromise plant growth. However, sufficient sugars to supply glycolysis coupled with fermentation to maintain NAD^+^/NADH homeostasis enable some plants to survive and grow without oxygen. This is typically observed in wetland plants, where starchy storage organs provide sugars to fuel metabolism and elongation growth to reach the water surface; and in seeds, where the endosperm reserves enable seedling establishment under flooded conditions. The higher glycolytic and fermentation rates in these conditions can lead to ATP levels that even surpass those of normoxic plants (Ishizawa et al. 1999; Summers et al. 2000).

However, many plant species are characterized by starchy and sugar rich storage organs or endosperms; however, only a few can mobilize these reserves effectively under oxygen-limiting conditions. In fact, systematic analysis among rice cultivars shows that anoxic seedling establishment is independent of reserve availability or levels of glycolytic or fermentation enzymes but is associated with high reserve breakdown rates coupled with ethanol fermentation (Magneschi et al. 2009). This result further highlights that the efficient mobilization of reserves is crucial to thrive under hypoxia.

**Figure 2.**
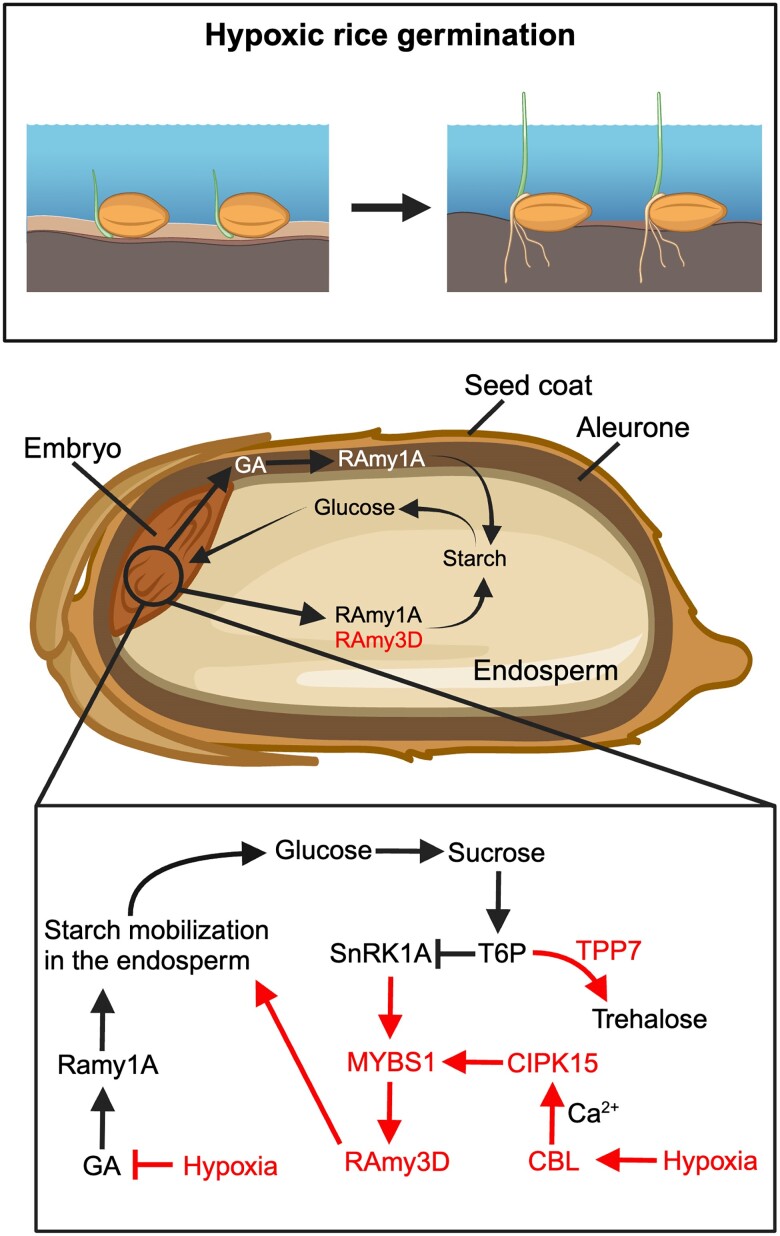
Molecular regulatory and metabolic pathways in rice during hypoxic germination. Low oxygen stress under submerged conditions leads to sugar starvation, which acts as a crucial upstream signal affecting metabolic regulation. Ca^2+^ functions as a secondary messenger to mediate downstream responses. CBL proteins bind to Ca^2+^ and interact with CIPK15, activating its kinase activity. This activated CIPK15 then interacts with SnRK1A, an upstream kinase of the transcription factor MYBS1, enhancing its activity and subsequently increasing αAmy activity for starch degradation in seeds. Seed imbibition triggers GA biosynthesis in the embryo. GA induces the αAmy gene. Concurrently, low oxygen-induced OsTPP7 relieves the inhibition of SnRK1A activity by T6P. OsTPP7 enhances the embryo axis–coleoptile's sink strength by converting T6P to trehalose. This lowers the T6P/sucrose ratio and promotes starch mobilization for energy production to facilitate coleoptile elongation. Low oxygen conditions shift aerobic respiration to anaerobic fermentation, inducing the expression of essential components, such as PDC and ADH. Created in BioRender. Triozzi et al. (2024)BioRender.com/g66n236.

In cereal grains, starch is usually the main reserve. A range of enzymes mediate starch degradation; however, α-amylase is key since it initiates starch hydrolysis (Fig. 2). In aerobic germination, gibberellins (GAs) are the main triggers for α-amylase gene expression, leading to efficient starch degradation to fuel aerobic respiration and enable germination (Hong et al. 2012; Yu et al. 2015). However, oxygen is required for the biosynthesis of GAs (Iacopino and Licausi 2020; Gómez-Álvarez and Pucciariello 2022), making the activation of GA-dependent α-amylases impossible under hypoxia or anoxia. Unlike many other cereals, which fail to germinate under anoxia, rice can induce a subfamily of GA-independent α-amylase genes by low O_2_ and sugar starvation (Loreti et al. 2003), which thus provide substrates for glycolysis coupled with fermentation to fuel anoxic rice seedling establishment.

Since sugar signaling feeds back on amylase activity, it plays a key role in regulating the mobilization rate of resources. In fact, rice accessions that excel at anaerobic seedling establishment have been found to adjust the sugar- and energy-sensing machinery (see Box 2) through the induction of a trehalose 6-phosphate phosphatase (OsTPP7), which removes trehalose-6-phosphate (T6P) (Kretzschmar et al. 2015). The removal of the inhibitory action of T6P on SnRK1A (see Box 2) enables the strong starvation signal provided by SnRK1 to activate and maintain a signaling cascade toward α-amylase. This creates a supply of sugars to fuel glycolysis and growth. The manipulation of CIPK15, which is a key regulator in the SnRK1 pathway, highlights the importance of starvation signaling to maintain a strong supply of sugars from storage, which is also important in mature elongating plants (Lee et al. 2009; Ye et al. 2018). Interestingly, seedling establishment also requires considerable anabolic activity for growth, which is often mediated by TOR signaling (Li et al. 2015). How seedling development coordinates the requirement of both catabolic and anabolic activities is still unclear, but the spatial separation of signals and corresponding regulation of transport between tissues is likely important.

Box 2. **Energy sensing in plants.**

The carbon and energy signaling networks consist of the plant kinase complexes Target of Rapamycin Complex 1 (TORC1) and SNF1-Related Kinase 1 (SnRK1) (Baena-González and Hanson 2017). Additionally, a set of C/S1 bZIP transcription factors are key players in the energy regulatory network (Dröge-Laser et al. 2018). The evolutionary conserved TORC1 and SnRK1 act antagonistically. Under conditions of high energy, the carbon and nutrient availability TORC1 is active and stimulates protein synthesis, growth, and development while suppressing catabolic processes such as autophagy. In contrast, SnRK1 is active during periods of starvation and suppresses energy-consuming processes and promotes catabolism (Baena-González et al. 2007; Polge and Thomas 2007). SnRK1 and TORC1 form a close-knit hub of regulators, which are crucial for cellular energy homeostasis, and the genetic removal of these pathways is often lethal or detrimental for growth (Menand et al. 2002; Baena-González et al. 2007).

Plants also produce T6P, an important sugar-signaling molecule, at very low concentrations. T6P levels tend to follow sucrose availability and are thus intricately linked with energy signaling. T6P in fact binds directly to SnRK1 to prevent phosphorylation and thereby inhibit SnRK1 activity (Zhang et al. 2009; Zhai et al. 2018). This ensures that starvation responses cannot be activated under high sugar conditions. T6P is synthesized by TPS1, which is located in the vasculature and below the shoot apical meristem (Fichtner et al. 2021). The emphasis of T6P might lie in source-sink coordination and whole plant integration of resources rather than cellular homeostasis (Fichtner and Lunn 2021).

Also, the induction of hypoxia responsive gene (HRG) expression relies heavily on sugar and energy signaling (Cho et al. 2021). Arabidopsis seedlings fail to maintain *ADH* induction under anoxic conditions unless they are supplied with external sucrose (Loreti et al. 2005, 2018). In Arabidopsis, TOR enhances RAP2.12 activity through the phosphorylation of 2 serine residues situated at the C terminal of several ERFVIIs (Fig. 1; see Box 1), resulting in stronger HRG expression (Kunkowska et al. 2023). This ensures that HRGs are most strongly expressed under submergence only when there is sufficient substrate and are instead inhibited under sugar starvation (Kunkowska et al. 2023).

In contrast, SnRK1A appears to interact with the promoters of *ADH1* and *PDC1* in Arabidopsis, where an active form of SnRK1A is required to stimulate HRG transcription (Cho et al. 2012). Similarly, in maize root tips, higher glucose availability dampened upregulation of HRGs during hypoxia (Sanclemente et al. 2021). Hypoxic gene expression is thus under tight control, and the right expression dosage is probably crucial for plant performance and integration with the metabolic demands of the cell (Dalle Carbonare et al. 2023).

Taken together, these observations provide both stimulation and suppression of HRGs by sugar and energy signaling. This might reflect variation in the cellular requirements of different cell types and developmental stages, such as storage, senescing, or proliferating cells. The multitude of factors affecting HRG expression, such as retrograde signaling (Meng et al. 2020) and calcium signaling (Fan et al. 2023), likely enables the regulatory flexibility in HRGs responses to sugars.

## Metabolic acclimation without sugars

A strong supply of carbohydrate reserves clearly aids performance under flooded and low oxygen conditions. However, many plants have a limited supply of reserve carbohydrates, and for many annual species this is only sufficient for the night period (Smith and Stitt 2007). Therefore, within a relatively short time, most species will run out of substrates during a flooding event, as seen in Arabidopsis and numerous wetland species (Van Veen et al. 2014, 2024; Loreti et al. 2018). Under limited substrate conditions, cells use autophagy to control the disassembly of organelles without compromising their function. During autophagy, cytosolic entities and organelles are engulfed by double membrane structured autophagosomes, which deliver their cargo to the vacuole for further degradation (Avila-Ospina et al. 2014; Otegui 2018). This enables the vacuole to become a source of amino acids and fatty acids, which can then be further catabolized, mostly through beta oxidation or entry into the TCA cycle (Baker et al. 2006; Hildebrandt et al. 2015; Fan et al. 2017; Heinemann and Hildebrandt 2021). Additional independent pathways for controlled organelle breakdown concern, first, the small senescence-associated vacuoles with lytic capacity to which chloroplast material is transferred. Second, chloroplast vesiculation (CV) is where the CV protein is targeted to the chloroplast to trigger budding of vesicles that contain the chloroplast content (Otegui et al. 2005; Wang and Blumwald 2014). Unsurprisingly, autophagy has been observed during flooding in Arabidopsis independent of its role in aerenchyma formation (Chen et al. 2015; Dalle Carbonare et al. 2023).

Autophagy is highly dependent on the energy- and nutrient-sensing pathways (see Box 2), where SnRK1 acts as an activator and the TORC1 complex acts as an inhibitor of autophagy to ensure activation only under starvation conditions (Soto-Burgos and Bassham 2017). Autophagy occurs frequently in normally developing plants, and starvation at the end of each night has also been reported (Izumi et al. 2013). During strong hypoxic conditions, the resources yielded by autophagy are of minimal use, since the TCA cycle and beta-oxidation require oxygen for their operation. In fact, the importance of autophagy during strong hypoxic conditions is unclear.

Carbon starvation under normoxic conditions has a characteristic metabolic profile with increased levels of branched chain amino acids that are supplied by autophagy (Usadel et al. 2008; Law et al. 2018; Barros et al. 2023). However, hypoxia-treated tissues sometimes show such patterns (Sato 2002; António et al. 2016), but sometimes do not (Narsai et al. 2011; Mustroph et al. 2014a). Overall, this indicates that despite activation and repression of SnRK1 and TOR, respectively, access to alternative substrates to sugar is not always feasible or under additional regulatory control during hypoxia.

However, when shoot tissues are submerged under dark conditions, oxygen tends to settle around 6 kPa (Herzog et al. 2023). When combined with continued slow but steady oxygen diffusion from the flood water, this enables plants to tap into the alternative resources provided by autophagy. Transcriptome profiling of submerged Arabidopsis pathways shows the strong activation of beta-oxidation and the catabolism of C-rich amino acids such as branched chain amino acids (Van Veen et al. 2016). Similar results have been obtained for rice, where also the characteristic increase in amino acid levels fueled by organelle breakdown were observed (Locke et al. 2018). In fact, mutants defective in autophagy have shown seriously impaired performance when submerged (Chen et al. 2015).

Organelle breakdown is highly coordinated and does not have to lead to cell death and is thus useful to bridge brief periods of starvation, including short flood events. However, extended periods of darkness or starvation combined with the characteristic accumulation of ethylene during flooding also trigger senescence, starting in the oldest leaves and gradually moving on to the youngest leaves (Rankenberg et al. 2024). Although physiologically not always necessary, during senescence the tissue is destined to die through complete cellular disassembly. Activation of senescence is not particularly beneficial, especially for sublethal flood durations, as demonstrated in Arabidopsis mutants with delayed senescence responses but intact autophagy pathways (Rankenberg et al. 2024). However, senescence is very common during flooding and is also observed in highly flood-tolerant species. Under normal conditions senescence is essential to repurpose building blocks, especially nitrogen (Guiboileau et al. 2012; Schippers et al. 2015).

The strong catabolic requirements of carbon backbones during a flooding event would indicate that flooded plants are more limited by carbon and not nitrogen for their survival. Indeed, ammonia, nitrate, and/or total free amino acids increase during flooding in *Rumex* species and rice (Sato 2002; Van Veen et al. 2013; Locke et al. 2018). Interestingly, the glyoxylate and gluconeogenesis pathways are transcriptionally activated upon flooding (Van Veen et al. 2016), which provide an opportunity to scavenge and conserve the carbon from organellar breakdown and synthesize sugars. Transcriptional data suggest that instead of glycolysis, gluconeogenesis is induced upon flood events (Van Veen et al. 2016). Although energetically costly, this provides an opportunity to supply other prioritized plant organs with the required limiting resources, in line with the functional purpose of senescence (Guiboileau et al. 2012; Schippers et al. 2015).

**Figure 3.**
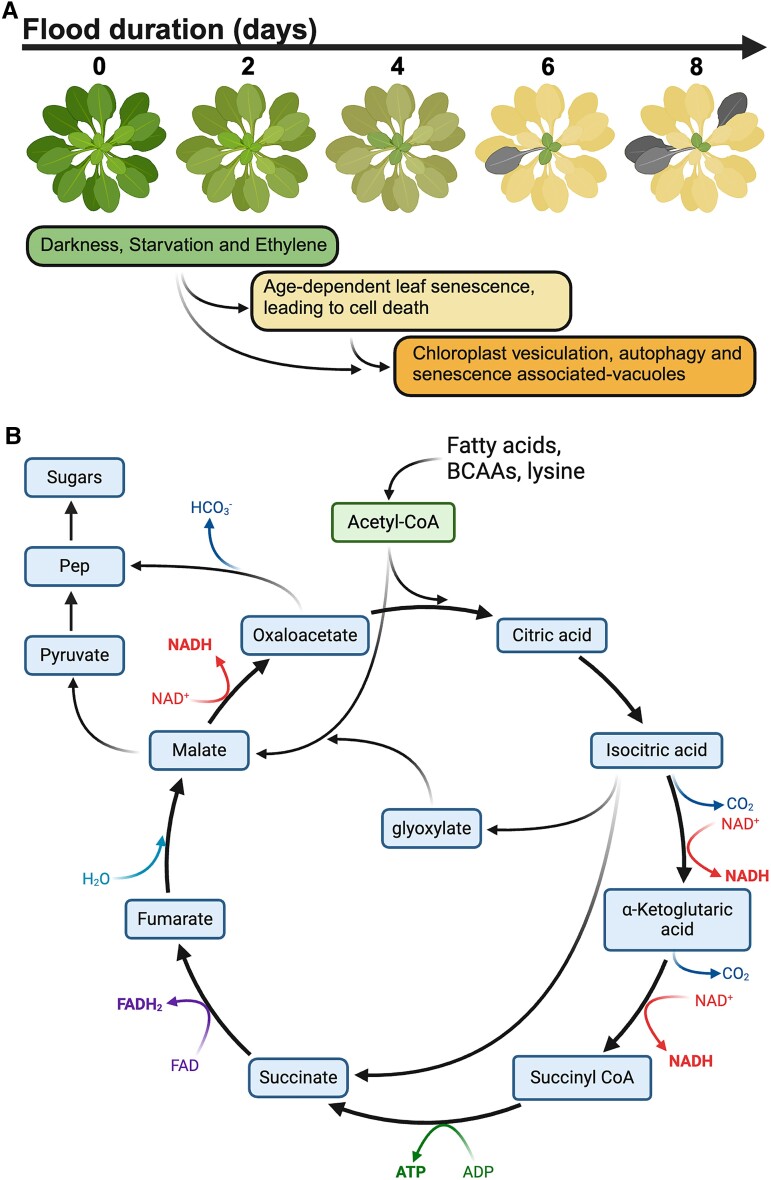
Long-term flood adaptations without a sugar supply. **A)** The sequential senescence phenotype of Arabidopsis completely submerged in the dark over the course of a week. The activation of the developmental program leads to death and controlled breakdown of organelles. **B)** Proposed metabolic route during flooding in dying leaves based on transcriptome and metabolome profiles. These findings are in line with a low abundance of sugars, which forces the cell to rely on alternative substrates. Created in BioRender. Triozzi et al. (2024)BioRender.com/z94e135.

In plants that do not heavily invest in easily accessible storage organs, such as many annual plants and crops, glycolysis and associated fermentation play a minor role in metabolism during flooding and reduced oxygen availability (Fig. 3). Indeed, the measurable ethanol production in Arabidopsis requires the addition of exogenous sugars (Santaniello et al. 2014). On the other hand, alternative substrates are essential for sustenance under submerged conditions (Chen et al. 2015). However, any functional significance of the senescence developmental program for whole plant performance and resource management would require functional transport of metabolites from the dying parts to prioritized tissues. Thus far it remains unclear to what extent transport takes place between organs and what is the functional significance of this process for long-term flood tolerance.

## Hypoxic metabolism under optimal growth conditions

Internal plant oxygen levels mostly result from diffusion from the atmosphere into plant tissues; hence, flooding and waterlogging significantly influence the occurrence of hypoxia in plants. However, oxygen is also produced through the oxygenic reactions of photosynthesis, which, together with oxygen consumption rates, mostly due to mitochondrial respiration, contribute to the final endogenous oxygen concentration in plants.

**Figure 4.**
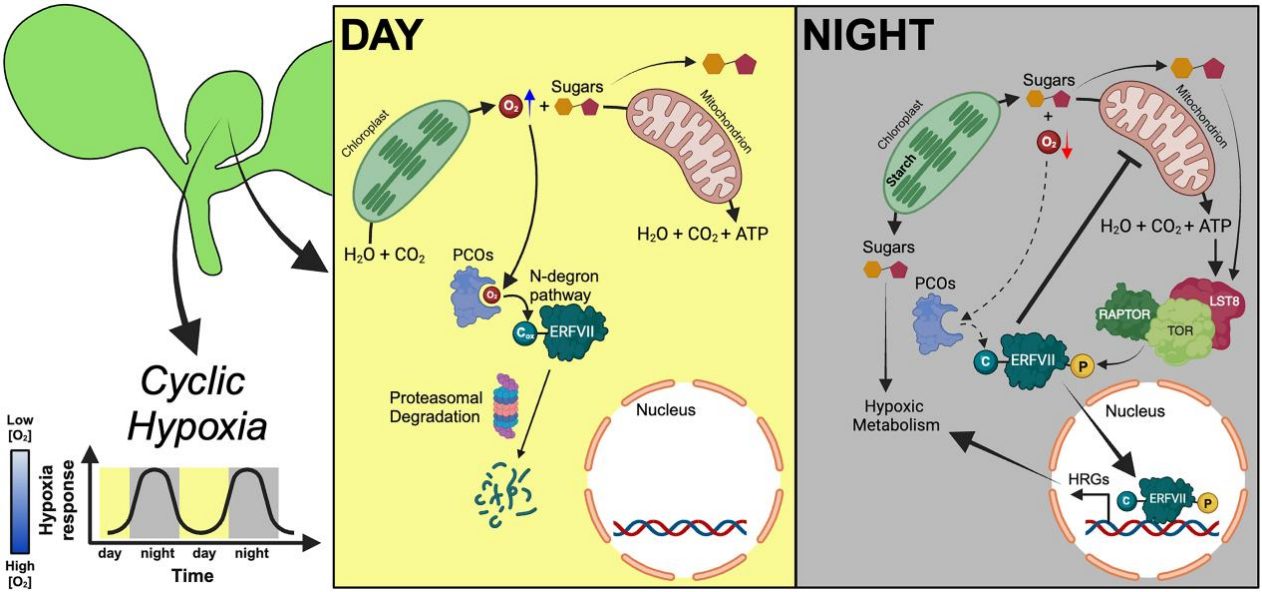
Cyclic activation of hypoxic metabolism in developing leaves. Young emerging leaves experience oxygen fluctuations under day/night cycles, which is referred to as cyclic hypoxia. This mechanism relies on ERFVII stability and activity. During the day, ERFVIIs undergo proteasomal degradation in young leaves through the N-degron pathway. However, an internal decreased in oxygen concentration occurs in young leaves at night because of the high respiration rate. Consequently, ERFVIIs are stabilized, translocate to the nucleus, and trigger the transcription of HRGs. This ERFVII-dependent cyclic mechanism creates a metabolic switch, which slows down the respiration rate in favor of the hypoxic metabolism at night. Cyclic hypoxia responses are also modulated by the activity of the energy sensor TOR complex, which depends on carbon and energy availability. Created in BioRender. Triozzi et al. (2024)BioRender.com/a44t283.

The transport of oxygen, as a component of the gaseous atmosphere, can also occur through the aerenchyma. However, hypoxic niches may form when the dense packing of cells or other barriers limit gas diffusion and/or when the cellular respiration rate is very high (Weits et al. 2021). This occurs in plants growing under otherwise optimal environmental conditions. Chronic hypoxic niches have to date been described in the shoot apex, lateral root primordia, and bulky fruits, which display varying degrees of hypoxia (Geigenberger et al. 2000; Van Dongen et al. 2003; Kerpen et al. 2019; Shukla et al. 2019; Weits et al. 2019; Xiao et al. 2021; Koo et al. 2024). Also crown galls and hormone-induced calli tend to develop a hypoxic microenvironment, and their growth requires the activation of the anaerobic metabolism. Besides the existence of hypoxic niches in normoxic plants, a recent study showed that oxygen fluctuations occur in young emerging leaves of Arabidopsis under day/night cycles, revealing the existence of cyclic hypoxia in plants (Fig. 4; Triozzi et al. 2024). Dynamic changes in oxygen concentration were previously reported in animal systems. Cyclic hypoxia is generated during tumoral growth where high cellular respiration rate, changes in red blood cell flux, vascular remodeling, and thermoregulation produce spatiotemporal oxygen dynamics (Michiels et al. 2016; Bader et al. 2020). Similarly, cyclic hypoxia in plants is generated by a fluctuating balance between oxygen production by photosynthesis and oxygen consumption by respiration in young leaves, thus promoting a homeostatic mechanism that controls the utilization of sugar reserves at night (Fig. 4; Triozzi et al. 2024). The daily establishment of cyclic hypoxia in young leaves is functional for plant growth, remarking the role of oxygen as a signaling molecule for plant growth and development (Weits et al. 2021). The mechanism underlying cyclic hypoxia could involve the TOR-dependent mechanism responsible for energy sensing and signaling under low oxygen conditions (Fig. 1; Kunkowska et al. 2023). Pharmacological inhibition of TOR activity was found to dampen cyclic hypoxia responses in young leaves at night (Fig. 4; Triozzi et al. 2024). Alternatively, the inhibition of TOR prevented energy consumption, thereby limiting high respiration rates and hampering cyclic hypoxia establishment.

Diffusion from oxygenic photosynthetic tissues, mostly through the aerenchyma, is an important source of O_2_ for many plant tissues in submerged plants (Herzog and Pedersen 2014). This suggests that cyclic oxygen levels may be widespread throughout the completely submerged plants lacking access to the atmosphere. Moreover, whether fluctuations in oxygen levels during the day/night cycle also occur in hypoxic niches such as the shoot apical meristem is still unclear. The development of in vivo oxygen sensors capable of monitoring daily oxygen fluctuations will be crucial to investigate when, where, and how oxygen dynamics occur in different plant tissues and organs.

Respiration rates and metabolic activity are actively regulated in response to declining oxygen levels (Geigenberger et al. 2000; Zabalza et al. 2009). Recently, the HYPOXIA-RESPONSIVE MODULATOR 1, which is a HRG and a target of RAP2.2, was found to attenuate mETC activity under hypoxic stress, leading to NADH accumulation and slowing down the TCA cycle, which redirects the metabolic flux toward the fermentation pathway (Tsai et al. 2023). Notably, the overexpression of an oxygen-insensitive version of RAP2.12 led to strong activation of ethanol fermentation and inhibition of cellular respiration, accompanied by higher levels of starch under sugar-repleting conditions (Paul et al. 2016). Similarly, lifetime imaging of NAP(D)H in young emerging leaves at night revealed how the shift away from oxidative phosphorylation was dependent on the ERFVII function (Triozzi et al. 2024). Although these findings provide examples of how ERFVII-dependent oxygen signaling could drive the metabolic flux toward glycolysis and fermentation, clarifying the intricate mechanisms that control the balance between aerobic and hypoxic metabolism requires further investigation and should consider the different plant developmental contexts and tissue-specific regulation.

The importance of hypoxic metabolism for plant growth is evident from mutants lacking fermentative enzymes ADH and PDC (*adh*, *pdc1xpdc2*), which were smaller than wild-type plants even when grown under aerobic conditions (Ventura et al. 2020). Additionally, the mutant lacking all 5 members of ERFVII family (*erfVII*), which is defective in the fermentative pathway, exhibited significantly impaired growth under normoxia (Triozzi et al. 2024). This indicates that hypoxic metabolism plays a role even in aerated plants. Why hypoxic metabolism needs to be activated even with sufficient oxygen and high energy yields is still unclear. Despite low yield, the advantage of glycolysis-driven ATP production is the incredibly fast rate of ATP generation that can be achieved with sufficient substrate. In contrast, the exploitation of the ATP yield of sugars with oxidative phosphorylation is highly efficient but relatively slow (Pfeiffer et al. 2001). Moreover, glycolytic intermediates are an important source of intermediates to support anabolism, and therefore proliferating cells rely heavily on glycolysis (Lunt and Vander Heiden 2011). Therefore, the young developing leaf tissue seems to mirror behavior of aerobic yeast that grow faster by rapidly consuming resources to leverage the high rate of glycolytic ATP generation despite aerated conditions (Rolland et al. 2002) and might even be instrumental for such proliferative cells to obtain a strong sink strength by virtue of high sugar requirements. However, it is very likely that hypoxic niches in aerobic plant tissues require metabolic adaptation in cells experiencing very localized hypoxia.

There is currently no clear explanation of the mechanism that establishes the trade-off between mitochondrial respiration and the fermentative metabolism, which both compete for the end-products of glycolysis. Regulation through the central metabolism arises out of a complex system of feedback and feedforward mechanisms and thermodynamic limitations. In these complex systems, the cellular demand has proven to be highly effective in controlling metabolic fluxes in order to satisfy the needs of the cell (Rohwer and Hofmeyr 2008; Sweetlove et al. 2010; Morandini 2013). Cellular identity and development, such as leaf and root initiation and growth, might be stronger drivers of metabolism during hypoxia and flooding than initially thought, highlighting the need to achieve a high spatiotemporal resolution.

To date there are only a few examples of plant cell–type-specific metabolism (Mira et al. 2019; Mira et al. 2023). This is mainly due to the inability to isolate sufficient amounts of tissue or groups of cells to analyze their metabolic activity and metabolites. A combination of single cell metabolomics (De Souza et al. 2020), mass spectrometry in vivo imaging (Horn and Chapman 2024), genetic-encoded biosensors (De Col et al. 2017; Steinbeck et al. 2020), and fluorescence lifetime imaging (Triozzi et al. 2024) offers a viable solution for mapping the spatiotemporal distribution and function of hypoxic metabolism during plant growth and development.

## Conclusions

From the reconfiguration of N metabolism, strong catabolism, and the controlled breakdown of organelles, to fermentation in the presence of oxygen, metabolic responses are highly dependent on the context of low oxygen availability. Consistent progress has been made in explaining the functional significance and regulatory mechanisms of the metabolic responses to hypoxia (**see Advances**). Although metabolite changes have been well characterized, the direction of metabolic fluxes is not always apparent. Exceptions are the clear start and endpoints of metabolism, such as starch and ethanol.

Isotope labeling is essential to reconstruct the precise direction of metabolic pathways, as is the case for N metabolism and the TCA cycle (António et al. 2016). However, isotope labeling has rarely been used to address metabolism under limited aeration conditions and should play a key role in explaining the integration of C and N metabolism, the destiny of organellar breakdown products, and transport capabilities during flooding.

Apart from identifying fluxes, understanding the control mechanism of the metabolic flow is crucial. The regulation of fermentation has been studied the most, but many questions still remain. Transcriptional HRGs induction can be sufficient to provide a fermentative flux, while sugar supply is also a major driver. In another paradox, the antagonists TORC1 and SnRK1 have both been reported to stimulate HRG induction. Fermentation can also be active because of hypoxia under high respiration and help to fuel rapid growth. Limited aeration results in a wide variety of metabolic adaptations. A clear understanding of the functional significance and control mechanisms will be critical in the development of flood-adaptive plants (**see Outstanding Questions**). Additionally, understanding the role of hypoxic niches and hypoxic metabolism under optimal growth conditions will help to further comprehend and modulate plant growth and development.

**Advances box**

- Under hypoxia, GABA metabolism allows for improved potassium homeostasis by desensitizing GORKs, which results in a lower steady-state membrane potential and antagonizes cell death.
- Senescence and the controlled breakdown of organelles provide catabolic substrates for flood tolerance.
- The magnitude of transcriptional activation of HRGs under hypoxia is strongly controlled by TORC1 and SnRK1 signaling, and transcriptional induction can be associated with either high or low energy and sugar availability.
- Cyclic hypoxia in young leaves demonstrates that spatiotemporal dynamics in oxygen availability exist on a daily basis, influencing plant growth.

**Outstanding questions box**

1. To what extent can and do metabolite transport processes play a role in integrating whole plant resource and redox management under hypoxia?
2. What are the driving forces orchestrating metabolic fluxes during flooding? How do these relate to cellular identity and developmental profiles?
3. Why is HRG induction usually increased by high sugar availability, while in some species the opposite is true? Is there a role for distinct cellular identity?
4. How widespread are cyclic fluctuations in oxygen and hypoxic metabolism throughout an individual plant and throughout the plant kingdom? Do they translate into differences in cellular metabolic activity?
5. What is the functional importance for plant cells to activate hypoxic metabolism under relatively adequate oxygen concentrations? How is the balance controlled between respiration and fermentation under mild hypoxia?

## Data Availability

No new data were generated or analyzed in support of this update.

## Dive Curated Terms

The following phenotypic, genotypic, and functional terms are of significance to the work described in this paper:

GORK Gramene: AT5G37500
GORK Araport: AT5G37500

adh1 Gramene: AT1G77120
adh1 Araport: AT1G77120

NAD CHEBI: CHEBI:13389

NADH CHEBI: CHEBI:16908

CIPK15 Gramene: AT5G01810
CIPK15 Araport: AT5G01810

CBL Gramene: AT3G57050
CBL Araport: AT3G57050
